# Beyond wellness: modeling the impact of organizational physical activity climate on job performance through psychological and behavioral pathways

**DOI:** 10.3389/fpsyg.2026.1817693

**Published:** 2026-05-20

**Authors:** Güven Dere, Selcan Ergun, Syed Ibrahim Bilal Majid, Ufuk Türen, Vladimir Simic, Lili Anna Hujber

**Affiliations:** 1 Department of Sports Management, İstanbul Rumeli Üniversitesi, İstanbul, Türkiye; 2 School of Foreign Languages, National Defence University, İstanbul, Türkiye; 3 Management Information Systems, OSTIM Technical University, Ankara, Türkiye; 4 Department of Industrial Engineering and Management, College of Engineering, Yuan Ze University, Taoyuan, Taiwan; 5 Department of Computer Science and Engineering, College of Informatics, Korea University, Seoul, Republic of Korea; 6 Faculty of Health and Sport Sciences, Széchenyi István University, Győr, Hungary

**Keywords:** organizational climate theory, organizational physical activity climate, physical activity behavior, job performance, job satisfaction, structural equation modeling, scale development

## Abstract

**Introduction:**

This study introduces Organizational Physical Activity Climate (OPAC) as a domain-specific extension of organizational climate theory, addressing the limited understanding of how workplace environments influence employees’ physical activity (PA), job satisfaction (JS), and job performance (JP). While prior research has primarily focused on individual-level determinants, organizational-level influences and their indirect effects on PA, JS, and JP remain insufficiently explored.

**Methods:**

Data were collected from 543 respondents through field visits to 60 universities and academic conferences in Istanbul. Exploratory and confirmatory factor analyses were conducted using JAMOVI-R, and structural equation modeling was performed using SmartPLS.

**Results:**

OPAC did not have a significant direct effect on job performance. However, PA partially mediated the OPAC-job performance relationship, while JS fully mediated this relationship. The serial mediation pathway from OPAC to PA, JS, and JP was also supported, although the effect was modest.

**Discussion:**

The study contributes to the literature by introducing and validating OPAC as a domain-specific organizational climate construct and by suggesting that its association with JP is primarily reflected through indirect behavioral and psychological pathways. It also offers practical implications for organizations aiming to enhance employee well-being and performance through health-supportive workplace climates.

## 1 Introduction

Technological advancement has dramatically reshaped modern life, simplifying daily tasks while simultaneously introducing new challenges—particularly the global rise of physical inactivity and its associated health risks. Since the Agricultural Revolution, human societies have moved toward structured, sedentary living, driven by labor specialization, food security, and centralized authority. These structural changes, accelerated by industrial and digital transformations, have reduced the physical demands of everyday life and promoted sedentary behaviors (Katzmarzyk and Lear, 2012).

This shift has created a mismatch between our evolutionary biology—adapted for mobility and endurance—and the physically inactive lifestyles prevalent today. The result is a rise in chronic diseases, musculoskeletal degeneration, and psychological disorders (Lee et al., 2012). Recognizing this, the World Health Organization has identified physical inactivity as a major public health concern linked to urbanization and industrialization (Roessler et al., 2012).

Promoting physical activity (PA) is now recognized as a global health priority. Research shows that regular PA reduces the risk of non-communicable diseases and improves cardiovascular, metabolic, and psychological outcomes (Warburton et al., 2006). Inactivity contributes significantly to the global burden of disease and strains healthcare systems (Lee et al., 2012). Sedentary work environments further exacerbate these risks, making PA promotion not only a public health issue but also a strategic concern for organizations.

Recent workplace health research also shows that physical activity promotion is being reshaped by new technological and organizational developments. Studies published after 2023 indicate a growing interest in the use of wearable and app-based tools to capture objective physical activity data in work settings, as well as in AI-assisted wellness systems that can personalize recommendations and monitor health-related patterns over time (Manger et al., 2025; Majumder and Dey, 2026). At the same time, post-pandemic scholarship has emphasized that workplace health promotion now operates in a changing environment shaped by hybrid work arrangements, redesigned organizational routines, and implementation challenges at the institutional level (Whitsel et al., 2023; Bhandari et al., 2026). These developments suggest that contemporary workplace wellness is becoming increasingly technology-enabled and context-sensitive. However, they also highlight the continued importance of understanding whether employees perceive their organization as genuinely supportive of physical activity, which remains insufficiently captured in the existing literature.

Regular PA is linked to a range of physical, mental, and social benefits (Warburton and Bredin, 2017). In occupational settings, these benefits extend to enhanced job satisfaction (JS) and job performance (JP) (Harter et al., 2003). However, while the general literature on organizational climate is well established (Anderson and West, 1998; Türen et al., 2014), the role of workplace climate in shaping PA behaviors remains underexplored.

Although prior studies have examined motivational climates in relation to exercise and academic performance (Digelidis et al., 2003), few have extended this inquiry to adult workplace contexts. School-based measures, such as the School Physical Activity Climate (SPAC) scale (Samuelson et al., 2010), offer valuable insights but lack transferability to occupational settings. Recent research underscores the relevance of workplace climate in shaping PA behaviors. For example, Tringali and Aldridge (2021) found that supportive PA climates enhance employee participation in activity programs. Similarly, Sonnentag and Pundt (2016) introduced a workplace exercise climate scale incorporating managerial values, organizational practices, and informal communication. However, these tools have limitations: Tringali and Aldridge (2021) rely on general health norms, and Sonnentag and Pundt’s model (Sonnentag and Pundt, 2016) lacks full alignment with core dimensions of organizational climate theory and does not provide a distinct, theoretically grounded construct specific to PA.

To address this gap, the present study introduces and operationalizes the concept of Organizational Physical Activity Climate (OPAC)—defined as employees’ individual perceptions of the extent to which their respective organization structurally and normatively supports PA. This research develops and validates a dedicated OPAC scale and investigates its relationship with employee PA, JS, and job performance. In doing so, the study contributes both theoretically—by advancing a novel, domain-specific framework—and practically, by offering a scalable tool to inform workplace health promotion strategies and performance enhancement efforts.

## 2 Background

### 2.1 Physical activity

PA refers to any bodily movement produced by skeletal muscle contractions that results in increased energy expenditure beyond resting levels. It includes routine behaviors such as shopping, cleaning, gardening, and stair climbing (Wilcox et al., 2002). PA is a multifaceted concept with definitions varying across disciplines. Caspersen et al. (1985) broadly defined it as all muscle-induced movement that expends energy, encompassing activities in domestic, occupational, transport, and leisure domains. Several scholars offer nuanced perspectives. Pate et al. (1995) emphasized joint and muscle engagement during everyday movements. Booth et al. (2012) defined PA as skeletal muscle contractions that raise energy expenditure above basal metabolic rate. Nystoriak and Bhatnagar (2018) highlighted physiological responses, such as increased heart rate, respiration, and fatigue.

Beyond its physiological dimension, PA is influenced by social and environmental factors. Biddle and Mutrie (2007) underscored the roles of gender, socioeconomic status, and social support, particularly within exercise settings. Similarly, Trost et al. (2002) found that parental and peer support are critical predictors of youth PA engagement. Given the conceptual variation, a clear and inclusive definition is essential. This study adopts an integrated view of PA as any voluntary movement driven by skeletal muscles that leads to measurable energy expenditure, occurring across daily living, work, transport, and recreational settings.

### 2.2 Physical activity and health

Extensive research highlights the substantial health benefits of PA, linking it to reduced risks of chronic illness and premature mortality. Regular PA helps prevent cardiovascular disease, certain cancers, hypertension, type 2 diabetes, and obesity, while also improving muscle and bone strength, blood pressure regulation, aerobic capacity, and motor skills. It is widely acknowledged as a key preventive measure against non-communicable diseases (Warburton et al., 2006), and the WHO has emphasized its protective role in combating heart disease, diabetes, and cancer. Beyond physical health, PA contributes to improved mental well-being, reduced symptoms of depression and anxiety, and enhanced social connectedness and cognitive performance (Eime et al., 2013). In contrast, sedentary behavior is strongly associated with increased health risks and early mortality (Fox et al., 2003). This body of evidence underscores the role of regular PA as a low-cost, widely accessible, and effective strategy for promoting both individual and public health.

### 2.3 Physical activity and work life

Work occupies a significant portion of adult life, especially in office-based roles characteristic of service and knowledge economies (Kalleberg, 2011). These settings are where organizational culture and managerial practices are most directly experienced. Increasing demands, competitiveness, and psychosocial stress in the workplace can lead employees to neglect physical and social needs, adversely affecting well-being and long-term performance (Bakker and Demerouti, 2007). While technology has improved efficiency, it has also reduced opportunities for PA. Sedentary work, compounded by ergonomic challenges and job stress, negatively impacts health, productivity, and job performance (Özer and ve Baltacı, 2008). Promoting PA has been linked to increased JS, better performance, and enhanced organizational outcomes (Bulut, 2013). However, integrating PA into daily work routines remains difficult, making communication about its importance a crucial first step.

Organizations that prioritize PA—through programs and social support—report improved employee health and productivity (Kim, 2018), as well as lower healthcare costs (Naydeck et al., 2008). Early evidence from Donoghue (1977) found that 50% of employees engaging in regular PA reported reduced stress and illness-related absences. Recent studies confirm these benefits. Rocha et al. (2024) observed improvements in health and mental well-being after a 12-week PA program, while Shrestha et al. (2018) and Ben-Ner et al. (2014) showed that interventions like treadmill desks reduce sedentary behavior without harming job performance. Industry reports also link corporate wellness programs to lower stress, absenteeism, and healthcare expenses (Pak et al., 1999).

Overall, consistent evidence shows that regular PA improves health, job performance, and retention (Naydeck et al., 2008), reinforcing the importance of cultivating a supportive climate for PA as part of a comprehensive organizational health strategy (Pronk and Kottke, 2009).

### 2.4 Organizational physical activity climate

*Organizational climate theory* defines climate as employees shared perceptions of formal and informal structures, practices, and cues that shape behavioral expectations within the workplace (Schneider, 1975; Reichers and Schneider, 1990). Climates may be general or domain-specific—targeting areas such as safety, service, or well-being. Across both types, recurring features such as leadership support, resource availability, communication clarity, and social norms influence how employees experience their environment (Patterson et al., 2005). Ostroff et al. (2003) emphasized that climate was not defined by a fixed set of dimensions, but by thematic clusters aligned with organizational goals—such as structural clarity, leadership support, psychological safety, and interpersonal relationships. These clusters have informed a range of strategic climate models, including those adapted for PA contexts. Although related, organizational climate and culture are conceptually distinct. Climate reflects the “what” of employee experience—perceptions of observable practices and procedures—while culture reflects the “why,” rooted in deeper organizational values and assumptions (Reichers and Schneider, 1990; Schneider et al., 2013).

The OPAC construct applies climate theory to the domain of PA. It captures employees’ perceptions of how organizational structures, leadership behaviors, peer norms, and environmental support enable or constrain PA participation during the workday (Schneider et al., 2013). These perceptions function as proximal behavioral determinants, shaping whether PA is seen as feasible, encouraged, and institutionally valued. In modern workplaces, PA is both a personal health behavior and a strategic organizational asset, linked to reduced absenteeism, enhanced job performance, and greater employee morale (Proper and van Mechelen, 2008). Yet, sustained participation depends more on environmental enablers—such as access to facilities, schedule flexibility, and managerial modeling—than on individual motivation (Sonnentag and Pundt, 2016). OPAC offers a structured framework to assess these conditions and to translate broad health goals into actionable organizational strategies.

Supportive PA climates have been associated with increased JS, employee engagement, and retention (Parks and Steelman, 2008; Conn et al., 2009). OPAC can stimulate individual-level behavior (PA) which can enhance work related individual level outcomes (JS and JP) by embedding wellness promotion into institutional routines and expectations. It also aligns with occupational health psychology’s shift from individual-focused interventions toward systemic, environmental strategies (Sorensen et al., 2004; Linnan et al., 2008) and corresponds with ecological health models that emphasize multilevel influences on behavior (McLeroy et al., 1988). Unlike symbolic wellness campaigns or narrowly targeted tools, OPAC provides a theory-driven, scalable framework suitable for diverse workplace contexts. Its focus on “physical activity” rather than “exercise” reflects a broader, more inclusive behavioral domain—encompassing actions like walking meetings, active commuting, and recreational movement. Ultimately, OPAC functions as a diagnostic model for evaluating whether organizations structurally enable and socially normalize PA. This study operationalizes OPAC through six interrelated dimensions—structural support, formal policy, managerial support, peer norms, communication, and opportunity—offering a conceptually grounded and practically applicable framework for advancing employee well-being.

### 2.5 Theoretical framework

This study draws upon four complementary theoretical frameworks to explain the mechanisms through which OPAC influences employee outcomes: Social Cognitive Theory, the Job Demands–Resources Model, Conservation of Resources Theory, and Organizational Climate Theory.

Social Cognitive Theory (Bandura, 1986) highlighted the reciprocal interaction between individual behavior, personal cognitive factors, and the social environment. In this context, OPAC represents an environmental influence that shapes employees’ PA behaviors. A climate that visibly supports PA may enhance employees’ self-efficacy—their belief in their ability to engage in PA at work. Observational learning is also relevant: employees are more likely to adopt health-promoting behaviors when they see peers doing the same. These mechanisms suggest that OPAC fosters PA, which, in turn, enhances JS and JP through improved well-being and psychological functioning.

The Job Demands–Resources Model (Demerouti et al., 2001) posits that all work environments comprise job demands and job resources. Job demands—such as workload and emotional strain—are associated with psychological costs, while job resources—such as managerial support, autonomy, and health-promoting climates—buffer these demands and enhance engagement. In this framework, OPAC functions as a job resource. By encouraging PA, it supports recovery, reduces strain, and enhances positive affective states, thereby contributing to higher JS and ultimately to improved JP (Bakker and Demerouti, 2007). This aligns with evidence showing that health-related job resources promote work engagement and performance.

Conservation of Resources Theory (Hobfoll, 1989) posits that individuals are motivated to acquire, maintain, and protect valued resources. PA enhances both physical and psychological resources, including energy, mood, and stress resilience. Empirical evidence supports these links: regular PA is associated with reduced fatigue (Puetz, 2006), lower stress, and greater JS (Conn et al., 2009). These outcomes may be partly driven by neurochemical changes, such as increased serotonin and endorphins (Hossain et al., 2024; Ren and Xiao, 2023). By facilitating resource gain and preventing depletion under high demands, OPAC contributes to employee sustainability and resilience, ultimately supporting JS and JP over time.

Finally, Organizational Climate Theory provides an overarching framework for understanding how OPAC shapes employee behavior and outcomes. Climate theory posits that shared perceptions of organizational policies, practices, and values significantly influence employee attitudes and actions (Schneider et al., 2013). OPAC reflects a domain-specific climate focused on PA. When employees perceive consistent organizational support—via formal policy, peer norms, and structural facilitation—they are more likely to engage in PA. These shared perceptions not only normalize PA but also foster psychological safety by signaling that prioritizing health will not result in stigma. This, in turn, enhances JS by aligning personal health goals with organizational values and improves JP through reduced stress, improved energy, and greater engagement. As a shared construct, OPAC reinforces the behavioral (PA) and psychological (JS) pathways that drive performance and productivity across the organization.

Together, these four theoretical frameworks position OPAC as a contextual enabler that facilitates resource access, behavioral reinforcement, and psychological alignment—ultimately promoting employee well-being and enhancing organizational performance.

#### 2.5.1 Organizational physical activity climate and job satisfaction

While exploring the concept of OPAC, we identified a notable gap in academic literature, as no studies to date have focused specifically on this construct. OPAC is often addressed only within the broader framework of organizational climate, which underscores the need for this study to clarify its definition by drawing on existing research related to organizational climate and JS.

Our literature review identifies various studies on organizational climate (Anderson and West, 1998; Bowen and Schneider, 2014; Türen et al., 2014), as well as research focused on motivational climates related to exercise and academic success (Digelidis et al., 2003). However, there remains a lack of focused studies on PA climate in organizations. Some research has shown a correlation between organizational climate and JS (Ahmad et al., 2018). Considering this information, the hypothesis is presented below.

*H_1_*: There is a significant and positive relationship between OPAC and employees’ JS.

#### 2.5.2 Organizational physical activity climate and job performance

As OPAC is a newly introduced construct developed in the present study, no prior research has directly examined its relationship with JP. To provide a theoretical foundation for this association, we draw on broader literature linking organizational climate to employee performance. For instance, Bhat and Bashir (2016) reported that a supportive organizational climate significantly enhanced teachers’ job performance. Similar positive associations between organizational climate and performance outcomes have been documented in other studies (Raza and Shah, 2010; Selamat et al., 2013). By positioning OPAC as a specific form of organizational climate, focused on promoting PA, we extend this line of reasoning to propose that a strong OPAC may similarly contribute to higher levels of JP. This leads to the following hypothesis:

*H_2_*: There is a significant and positive relationship between OPAC and employees’ JP.

#### 2.5.3 Job satisfaction and job performance

The link between JS and JP remains a foundational concern in organizational psychology (Landy, 1989). Early interest in this relationship gained momentum after the Hawthorne studies, which highlighted the importance of psychological and social factors in workplace productivity (Luthans, 2011). A landmark empirical contribution was made by Brayfield and Crockett (1955), who found no significant association between JS and JP but nonetheless laid the groundwork for future research. Over the years, studies have taken differing positions, with some suggesting that performance leads to satisfaction—owing to recognition and rewards (Siegel and Bowen, 1971; Sheridan and Slocum, 1975)—and others arguing that satisfaction motivates greater performance (Judge et al., 2001; Oh et al., 2014). Affective events theory (Weiss and Cropanzano, 1996) supports the latter, proposing that positive emotions foster improved work output.

Judge et al. (2001) reignited scholarly interest by conducting a comprehensive review that addressed earlier methodological inconsistencies and suggested a modest but positive association. This work served as a catalyst for further exploration. For example, Katebi et al. (2022) reviewed 913 studies and found a moderate, statistically significant positive relationship between JS and JP. Similar findings were reported in the Turkish context by Bozer and Yanık (2020) and Akburak et al. (2020), providing empirical support for a generalized JS–JP link across sectors and cultural settings.

In this study, JS is positioned as both an outcome of OPAC and a mediator between OPAC and JP. A climate that visibly supports PA may improve employee well-being and affective states, which in turn enhance motivation and job outcomes. Based on this theoretical and empirical groundwork, the following hypothesis is proposed:

*H_3_*: There is a significant and positive relationship between JS and JP.

#### 2.5.4 Mediating role of job satisfaction between organizational physical activity climate and job performance

Studies have shown significant associations between organizational climate and employee performance (Adeyemi, 2008; Bhat and Bashir, 2016), as well as the impact of PA on workplace outcomes. Engaging in regular PA improves mood, reduces fatigue, and supports psychological well-being, thereby enhancing both JS and JP (Puetz, 2006; Conn et al., 2009). While OPAC is a newly introduced construct, its conceptual foundation is consistent with prior research linking climate and PA-related outcomes. JS is widely regarded as a key predictor of JP. Judge et al. (2001) confirmed a positive association between the two, a finding reinforced by Katebi et al. (2022), who reported a moderate, statistically significant relationship. Higher JS is associated with increased motivation and engagement, which contributes to improved JP. These findings suggest that JS may mediate the effect of OPAC on JP. A climate supportive of PA can enhance JS by improving well-being, which in turn fosters stronger performance. This leads to the following hypothesis:

*H_4_*: JS mediates the relationship between OPAC and JP.

#### 2.5.5 Organizational physical activity climate and physical activity

OPAC, defined by the organizational emphasis on PA, support structures, and cultural encouragement, plays a key role in shaping employees’ weekly PA levels. “Booster Breaks” program exemplifies how promoting PA through brief, structured activities can positively shift workplace norms (Taylor et al., 2010). When PA is integrated into organizational policies and supported by leadership and peer norms, employees are more likely to participate, resulting in sustained physical and mental health benefits (Jirathananuwat and Pongpirul, 2017).

Empirical findings consistently link supportive organizational climates with higher PA levels. Tringali and Aldridge (2021) demonstrated that a positive PA climate promotes program participation, primarily shaped by shared norms rather than individual willpower. Singh et al. (2024) reinforced these insights in the large-scale “15 Minute Challenge”, where over 11,000 employees across two countries reported improved PA levels, fitness, sleep, and mood. Despite such evidence, prior studies lack a validated, theory-driven construct to assess PA climate. The present study addresses this gap by proposing OPAC—a structured, climate-based framework grounded in organizational climate theory. OPAC is hypothesized to influence employee PA by reinforcing shared norms, available resources, and behavioral expectations:

*H_5_*: There is a significant and positive relationship between OPAC and PA.

#### 2.5.6 Physical activity and job performance

Regular PA is widely acknowledged to benefit both individual health and JP. Increases in weekly PA levels have been positively linked to JS, work attendance, and overall performance. Anderson et al. (2009), in a meta-analysis, found that workplace-based PA interventions significantly improve attendance and reduce job-related stress, underscoring PA’s contribution to organizational outcomes. Similarly, Grabara et al. (2018), in a cross-sectional study of Polish teachers, reported a positive association between weekly PA and perceived job ability, especially among women. These findings align with broader evidence confirming that PA enhances JP (Jirathananuwat and Pongpirul, 2017). Frequent engagement in PA appears to support job capabilities and workplace efficiency. Based on this foundation, the following hypothesis is proposed:

*H_6_*: There is a significant and positive relationship between PA and JP.

#### 2.5.7 Mediating role of physical activity between organizational physical activity climate and job performance

A supportive organizational climate for PA may indirectly enhance JP by increasing employees’ weekly activity levels. Grossi et al. (2021) found that job control and expectations influence both PA and overall well-being, though PA played only a modest mediating role. In contrast, a systematic review by Jirathananuwat and Pongpirul (2017) confirmed that workplace-based PA interventions effectively increase PA levels. These findings imply that OPAC may positively affect JP indirectly by promoting PA. Thus, the following hypothesis is developed:

*H_7_*: PA mediates the relationship between OPAC and JP.

#### 2.5.8 Physical activity and job satisfaction

PA is considered positively associated with both psychological well-being and JS (Jirathananuwat and Pongpirul, 2017). Arslan et al. (2019) found that employees engaging in PA at least three times per week for an hour reported significantly higher JS and quality of life. Similarly, Romero-Carazas et al. (2024) showed that PA mediates the relationship between burnout and JS among nurses in Peru, suggesting that regular activity helps reduce burnout and enhance satisfaction. Other studies confirm that active employees consistently report greater JS and life quality than inactive peers (de-Pedro-Jiménez et al., 2021; Yan et al., 2022). Based on this evidence, the following hypothesis is proposed:

*H_8_*: There is a significant and positive relationship between PA and JS.

#### 2.5.9 Serial mediating role of physical activity and job satisfaction between organizational physical activity climate and job performance

The proposed serial mediation model draws upon four theoretical foundations. First, Organizational Climate Theory posits that shared perceptions, such as structural and normative support for PA, shape behavior, with a strong OPAC expected to promote PA engagement. Second, Social Cognitive Theory suggests that OPAC fosters a sense of efficacy and encourages healthy behavior through environmental modeling. Third, the Job Demands–Resources Model identifies both PA and JS as critical resources that buffer stress and enhance JP. Fourth, the Conservation of Resources Theory holds that regular PA builds physical and emotional resources, supporting JS and ultimately contributing to JP.

Empirical studies reinforce this theoretical logic. OPAC has been positively linked to PA participation (Arslan et al., 2019), while PA itself improves JS and JP (Yan et al., 2022). Conn et al. (2009) showed that workplace PA initiatives enhance health and performance. Yan et al. (2022) demonstrated that perceived physical competence boosts JS and JP. Dallmeyer et al. (2023) further confirmed the sequential pathway from OPAC through PA and JS to JP. Based on these theoretical foundations and empirical findings, the following hypothesis is proposed:

*H_9_*: PA and JS sequentially mediate the relationship between OPAC and JP.

**Figure 1:**
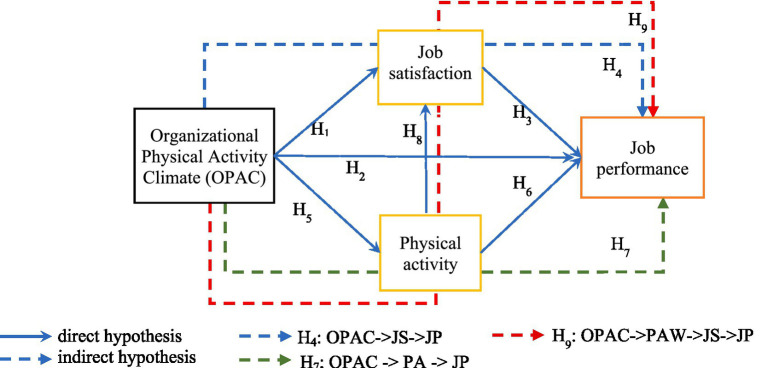
Research model.

The research model is presented in Figure 1.

## 3 Methodology

### 3.1 OPAC scale development

To develop the OPAC Scale, we followed the six-phase framework proposed by Kyriazos and Stalikas (2018) and DeVellis and Thorpe (2021). This structured process ensures methodological rigor and conceptual alignment in scale construction:

(1) *Generating the item pool*—A comprehensive 10-item pool was created through literature review and expert consultation, prioritizing conceptual coverage over parsimony. Items were crafted to express single, unambiguous ideas, adhering to grammatical clarity in Turkish. Drawing on existing measures—such as organizational climate scales (Thumin and Thumin, 2011; Türen et al., 2014), individual PA behavior assessments (Dallmeyer et al., 2023), the workplace exercise climate scale (Sonnentag and Pundt, 2016), and the SPAC scale (Samuelson et al., 2010)—we adapted principles relevant to the workplace setting. From Thumin and Thumin (2011), we adopted a perceptual approach to climate tied to actionable structures. Türen et al. (2014) emphasized strategic alignment, guiding our conceptualization of PA promotion as a climate issue. Dallmeyer et al. (2023) informed item phrasing through individual-level PA sensitivity, while Samuelson et al. (2010) and Sonnentag and Pundt (2016) contributed domain-specific conceptual foundations. Unlike prior tools, our focus was to capture the extent to which organizational policies, routines, and structures support PA in adult workplaces.
(2) *Theoretical framework*—Organizational Climate Theory views climate as shared perceptions of structures, practices, and cues within specific behavioral domains (Schneider, 1975; Reichers and Schneider, 1990). Climate constructs can be general or domain-specific, with core dimensions consistently emerging across the literature (Patterson et al., 2005; Schneider et al., 2013). In PA contexts, relevant dimensions include policy commitment, resource allocation, peer influence, communication, and access. Building on this foundation, we derived six thematic areas through literature synthesis and iterative classification, guided by frameworks from Ostroff et al. (2003), Zohar and Hofmann (2012), and others. The OPAC framework is built upon six core dimensions that collectively define the organizational climate for PA. *Structural support* refers to the tangible infrastructure and institutional arrangements that facilitate PA, such as access to facilities or equipment. *Formal policy* encompasses rules, procedures, and performance metrics that institutionalize PA as part of organizational practice. *Managerial support* captures leadership behaviors and signals that demonstrate commitment to encouraging PA among employees. *Peer norms* reflect informal expectations and observe behaviors of coworkers, shaping a socially reinforced climate. *Communication* involves the ways in which information, encouragement, and discourse about PA are conveyed within the organization. Finally, *opportunity* refers to the degree of temporal and procedural flexibility that enables employees to engage in PA during the workday. Together, these dimensions formed the conceptual scaffolding for item development and provide a comprehensive basis for evaluating PA climates in organizational settings, addressing the limitations of prior measures.
(3) *Review and critique of existing measures***—**Sonnentag and Pundt (2016) conceptualized Organizational Exercise Climate with three components—management values, practices, and communication. While their 12-item scale captures symbolic support (e.g., valuing exercise, peer respect), it omits structural elements such as policy integration and logistical enablers. Samuelson et al. (2010), in contrast, developed the SPAC scale for educational settings, emphasizing infrastructure and policy mandates. However, it lacks transferability to workplace contexts and overlooks key perceptual and social dimensions such as peer norms and time flexibility. These gaps highlight the need for a comprehensive and contextually adaptable instrument. The OPAC scale was created to meet this need by incorporating structural, symbolic, procedural, and social dimensions applicable across diverse organizational environments.
(4) *Conceptualizing and item-level justification for OPAC scale***—**In response to the identified gaps, this study introduces OPAC as a novel construct capturing the workplace climate for PA. Unlike prior measures focused narrowly on “exercise,” OPAC adopts a broader view of PA, encompassing both structured and unstructured behaviors (e.g., walking, biking, recreational sports) in line with ecological health models. The scale’s items were designed to reflect key dimensions of organizational climate theory while addressing limitations in existing tools. As shown in Table 1, OPAC1 and OPAC2 uniquely position physical fitness as a formal performance concern—an advancement over earlier instruments (Samuelson et al., 2010; Sonnentag and Pundt, 2016), which do not institutionalize PA through HR or appraisal systems. OPAC3 expands support beyond general “training” by highlighting access to expert advisory services, reinforcing structural and communicative dimensions of climate. OPAC4 addresses time autonomy, an often-overlooked facilitator of PA. OPAC5 and OPAC6 differentiate between competitive and non-competitive sport encouragement, reflecting motivational diversity and adapting concepts from Samuelson et al. (2010), originally applied in educational settings. OPAC7 generalizes Samuelson’s “activity bus” model to the workplace by introducing logistical facilitation, such as transportation support. OPAC8 captures peer norm influence by referencing coworkers’ behavioral engagement, reflecting socially embedded expectations. OPAC9 focuses on facility provision, adapting Samuelson et al.’s (2010) structural items for general organizational contexts. OPAC10 extends the range of supported activities to include outdoor and alternative options (e.g., hiking, cycling), broadening Samuelson’s route-based supports into a wellness-oriented infrastructure framework. Together, the OPAC items integrate symbolic, structural, and procedural aspects of PA climate, offering a formalized and scalable tool for workplace assessment and intervention.
(5) *Specify the format for measurement***—**After reviewing the item pool for construct alignment and redundancy, minor revisions were made through internal consensus. All items were then formatted using a five-point Likert scale ranging from 1 (“Strongly Disagree”) to 5 (“Strongly Agree”), applied uniformly across all scales in the survey instrument.
(6) *External review of the item pool***—**To ensure face validity—i.e., the extent to which items appear to measure the intended construct (Haynes et al., 1995)—six experts in organizational behavior and Turkish language reviewed the item pool. They assessed clarity, relevance, and grammatical accuracy. Based on their feedback, three items were revised for linguistic clarity; no items were removed, and no redundancy was noted. The review confirmed that the items were clear, well-aligned with the OPAC construct, and appropriately worded.

**Table 1:** Structure of construct with justifications.

Code	Item	Dimension	Justification
OPAC1	In performance evaluations, employees’ physical fitness is taken into account.	Formal policy	Clearly positions physical activity as a formal performance criterion, which elevates its importance in employee accountability structures—absent in both Sonnentag and Pundt (2016) and Samuelson et al. (2010).
OPAC2	Physical fitness has equal weight compared to other performance criteria.	Formal policy	While parity between physical fitness and other performance metrics exists in educational contexts (e.g., Samuelson et al. (2010)), OPAC2 extends this principle to organizational policy. It signals formal commitment to health by embedding fitness into core performance criteria- absent in Sonnentag and Pundt (2016).
OPAC3	There are qualified experts assigned to advise me on improving my physical fitness	Structural support and communication	Improves upon generalized ‘training’ references in Sonnentag and Pundt (2016) by specifying advisory access to physical fitness experts.
OPAC4	My job duties do not prevent me from allocating time to improve my physical performance.	Communication and opportunity	Highlights realistic time flexibility—an often overlooked but critical enabling conditions in climate measures—absent in both Sonnentag and Pundt (2016) and Samuelson et al. (2010).
OPAC5	Competitive sports events are encouraged.	Managerial support	Reframes interscholastic event participation in Samuelson et al. (2010) to fit proactive support for competitive activities in workplace settings. This is also used in Sonnentag and Pundt (2016).
OPAC6	Non-competitive sports activities are encouraged.	Managerial support	Adds inclusivity via non-competitive, recreational opportunities—broadening from Samuelson et al.'s (2010) school-based focus and Sonnentag and Pundt (2016).
OPAC7	When needed, transportation is provided by my organization to support participation in sports.	Opportunity and structural support	This item addresses logistical barriers by adapting Samuelson et al.’s (2010) “activity bus” concept for adult commuting and access. Like the “organizational discounts in fitness centers” noted by Sonnentag and Pundt (2016), it represents a passive and common form of support—though such memberships seldom lead to actual exercise.
OPAC8	My colleagues exercise for at least 3 h a week on average.	Peer norms	Adds a behavioral norm anchor by quantifying peer activity—offering a socially embedded signal of climate expectations- absent in both Sonnentag and Pundt (2016) and Samuelson et al. (2010).
OPAC9	Necessary sports facilities and spaces are made available for employees’ use.	Structural support	Aligns with Samuelson et al.’s (2010) ‘facility access’ items but adapted to non-educational workplace contexts. It reflects a realized organizational investment in PA—an element notably absent in Sonnentag and Pundt’s (2016) operationalization.
OPAC10	My organization supports alternative sports activities such as cycling, walking, hiking, or mountaineering.	Structural support	Expands the concept of structural support to include alternative and nature-based activities (e.g., hiking, mountaineering), which are underrepresented in workplace-focused instruments. While Samuelson et al. (2010) include support for walking/biking routes to school, OPAC10 generalizes and extends this to recreational and wellness-focused activity infrastructure.

### 3.2 Pilot study

To avoid inflated estimates, a larger pilot study was conducted. Following Teare et al. (2014), the OPAC Scale was administered to 80 academics in Istanbul via convenience sampling. The goal was to assess initial psychometric properties using explanatory factor analysis (EFA), item-total correlations, Cronbach’s alpha, and Spearman’s rho, given non-normal data. As shown in Table 2, KMO exceeded the 0.60 threshold and Bartlett’s test was significant (*p* < 0.05), confirming suitability for factor analysis. Cronbach’s alpha values were above 0.70, and all corrected item-total correlations exceeded 0.20 (Streiner and Norman, 2008). Items OPAC4, OPAC5, and OPAC10 were excluded due to high uniqueness or cross-loadings. The final scale retained seven items with significant positive inter-item correlations.

**Table 2:** Results of EFA, Cronbach’s *α*, Correlation Analysis, and Corrected Item-Total Correlations of the Pilot Study (OPAC).

Item ID	Sub-sample-1 (*n*_1_ = 40)	Sub-sample-2 (*n*_2_ = 40)	Factor loadings	Correlation analysis (Spearman’s rho)						
				1	2	3	4	5	6	7
OPAC1	0.731	0.621	0.753	1						
OPAC2	0.479	0.558	0.682	0.502**	1					
OPAC3	0.607	0.646	0.777	0.474**	0.474**	1				
OPAC 6	0.734	0.499	0.548	0.392**	0.336**	0.377**	1			
OPAC 7	0.650	0.622	0.752	0.419**	0.271*	0.502**	0.399**	1		
OPAC 8	0.599	0.460	0.617	0.354**	0.329**	0.308**	0.463**	0.385**	1	
OPAC 9	0.747	0.528	0.685	0.418**	0.251*	0.281*	0.263*	0.264*	0.534**	1

*p < 0.05, **p < 0.01, ***p < 0.0010. KMO: 0.800; BTS: χ^2^ = 129, *p* < 0.000; Cronbach’s α: 0.798.

#### 3.2.1 Assessing the reliability and validity of the pilot study

After the item pool is developed, examined, and applied to the research sample as previously determined, the next step is to examine and evaluate the items individually. This stage, along with the development of the items, can be described as the heart of scale development. A strong correlation between the items indicates that the item is reliable, and the reliability of the item indicates that the scale is reliable. EFA and correlation analysis (CA) were applied to test the validity and reliability of the scales in the pilot study. The sample was first checked to be suitable for EFA by using KMO and Bartlett’s tests. According to the test results (*KMO_OPAC_* = 0.826; *p_OPAC_* < 0.000), the samples are proper for EFA (Hair et al., 2014) at meritorious and middling level successively. Most factor loadings exceed 0.70, and there is a greater than 0.1 variation between factor loadings. For the factor analysis, loadings over 0.70 are seen as a sign of a clearly defined structure. Additionally, since the visual inspection reveals a significant number of inter-correlations of variables in the factors of the scales greater than 0.30, factor analysis can be considered appropriate (Hair et al., 2014).

#### 3.2.2 Optimize scale length

A scale’s Cronbach’s alpha is influenced by both item intercorrelations and the number of items. While shorter scales encourage participation, they may compromise reliability. Thus, a balance between brevity and internal consistency is essential. When sample size permits, it is advisable to divide the sample into two random subsamples and compare alpha values. If both subsamples were drawn under similar conditions, their Cronbach’s alpha scores and related analyses should be comparable. In such cases, items with consistently high reliability across subsamples are retained, while those with consistently low values are removed (DeVellis and Thorpe, 2021). In this study, the sample was split into two subsamples, and Cronbach’s alpha values were computed for each scale. Table 2 presents the corrected item-total correlations across both groups. As shown in Table 2, all corrected item-total correlations exceed the 0.20 threshold (Streiner and Norman, 2008) and exhibit similar patterns across subsamples. Based on these results, seven items were retained for the OPAC scale prior to the main data collection.

### 3.3 Other scales

(i) *Job satisfaction*—A five-item scale proposed and used by Judge et al. (2000) based on Brayfield and Rothe (1951) and adapted by Keser and Bilir (2019) is employed. The scale is designed with a 5-level Likert-type type. The scale showed acceptable internal consistency, with a Cronbach’s alpha of 0.85.
(ii) *Job performance*—This construct was measured using a five-item self-evaluation scale originally developed by Sigler and Pearson (2000) and later adapted to Turkish by Çöl (2008). Respondents rated themselves on a seven-point Likert scale ranging from 1 (strongly disagree) to 7 (strongly agree). The scale assesses five aspects: overall performance, interpersonal effectiveness, timeliness, work quality, and goal achievement. The scale showed acceptable internal consistency, with a Cronbach’s alpha of 0.76 (Çakıcı and Doğan, 2014).
(iii) *Physical activity*—This variable was measured using a single-item question on the weekly frequency of intensive PA, workout, or exercise. This approach has been used in prior studies (Engberg et al., 2015; Zhang et al., 2025) and is appropriate when the aim is to capture a concrete behavioral fact, such as frequency of engagement, rather than a broader multidimensional psychometric construct (Rossiter, 2002; Bergkvist and Rossiter, 2007). Prior measurement research also supports the use of single-item indicators for clearly defined constructs (Robins et al., 2001; Wanous et al., 1997). Specifically, in the PA literature, brief PA frequency measures have shown reasonable validity compared with longer instruments (Milton et al., 2011). In addition, brief measures reduce response burden and may help limit fatigue-related declines in response quality, particularly in studies with multiple constructs (Rolstad et al., 2011; Galesic and Bosnjak, 2009). This is consistent with satisficing theory, which suggests that fatigued or less motivated respondents are more likely to provide less effortful responses (Krosnick, 1991; Krosnick and Presser, 2010).

However, because this measure captures only frequency, it does not reflect other dimensions of PA, such as duration, type, or intensity variation (Liu et al., 2025; Sallis and Saelens, 2000). Therefore, PA-related findings should be interpreted cautiously as reflecting general activity frequency rather than comprehensive physical activity behavior.

### 3.4 Population and sampling

The high prevalence of sedentary behavior and its health risks among academic professionals is well-documented (Ghosh et al., 2023). Given consistent calls for PA interventions targeting this group, academics were selected as the study population. Data were collected from academic staff in Istanbul, Türkiye’s most populous province and home to 60 universities (İstanbul Valiliği, 2021). In 2022–2023, Istanbul’s academic personnel numbered 40,140 was obtained from the YÖK Statistics Portal by retrieving province-based higher education statistics for Istanbul for the 2022–2023 academic year (Yükseköğretim Kurulu Başkanlığı (YÖK), 2023). Using Cochran’s formula (Cochran, 1977) with a 95% confidence level and ±2.5% margin of error, the minimum required sample was 381. The achieved sample size of 543 exceeds this threshold, providing sufficient statistical power. However, as participation was based on voluntary responses rather than probability-based selection, the sampling approach was deemed as non-probabilistic convenience sampling.

### 3.5 Data collection

Data was collected from academic personnel via in-person and digital methods. Researchers visited 60 universities in Istanbul and engaged academic staff during conferences and faculty meetings. Additionally, survey links were distributed through academic email groups, reaching 5,500 addresses. Data collection occurred between May 5, 2023, and November 17, 2024, yielding 543 valid responses (response rate: 9.8%). Given this response rate, the possibility of nonresponse bias cannot be excluded, as individuals with a higher interest in physical activity or health-related issues may have been more likely to participate. However, to overcome this limitation, data were collected across multiple universities, disciplines, and academic ranks, aiming to capture heterogeneity within the academic population. However, as Table 3 summarizes the sample, regarding PA, 54.1% engage regularly, and 45.9% do not which considerably reduces the chances of non-response bias. The most common PA frequency is 3 days/week (21.7%).

**Table 3:** Descriptives.

Variable	*μ*	*σ*
OPAC	2.47	0.91
JS	3.43	0.89
JP	5.84	0.97
Age	47.8	7.88

Variable		f	%
Gender	Female (0)	236	43.4
	Male (1)	307	56.6
Academic rank	Full Professor	88	16.2
	Associated Professor	200	36.8
	Assistant Professor	255	47.0
Regular PA	Yes	294	54.1
	No	249	45.9
PAW	0	128	23.6
	1	55	10.1
	2	95	17.5
	3	118	21.7
	4	75	13.8
	5	43	7.9
	6	18	3.3
	7	11	2.0

*n* = 543; f, frequency; PA, Physical Activity; OPAC, Organizational Physical Activity Climate; JS, Job Satisfaction; JP, Job Performance; PAW, Physical Activity/Week.

In the data collection process, participants were not asked to report the names of their universities. This approach was adopted for ethical reasons, as universities did not agree to be identifiable in the study, and also to promote more valid responses by minimizing concerns related to social desirability bias. For this reason, this study does not permit comparisons across universities or the examination of shared climate perceptions at the organizational level, and OPAC was therefore treated as an individual-level perception of the organizational environment.

## 4 Analyses and results

This section presents the results related to scale validity and reliability, including outputs from EFA, SmartPLS outer loadings, average variance extracted (AVE), HTMT ratios, Cronbach’s alpha (‘*α*’), composite reliability (‘*c*’), Spearman correlations, and finally, the path coefficients along with their corresponding *p*-values.

### 4.1 Scale validity

The validity of our scales was checked by conducting EFA, CFA, and convergent/discriminant validity analyses. The reliability of our scale was examined by performing internal consistency and CR analyses. Table 4 also displays the results of the outer loadings obtained from SmartPLS.

**Table 4:** Outputs of exploratory factor analysis and outer loadings.

Variable	JAMOVI-R						SmartPLS				
	Factor desc. statistics		EFA				Outer loadings	AVE	HTMT–Matrix		
	mean	SD	Factor loadings	(%) Variance	KMO test	Bartlett’s test (*p*)			1	2	3
OPAC (7-item)	2.47	0.911	0.675–0.755	48.9	0.835	0.001	0.599–0.772	0.648	-		
JP (5-item)	5.84	0.966	0.705–0.878	66.1	0.848	0.001	0.764–0.854	0.621	0.451	-	
JS (5-item)	3.47	0.850	0.741–0.829	62.4	0.801	0.001	0.749–0.820	0.489	0.166	0.324	-

*n* = 543, HTMT, Heterotrait-Monotrait Ratio.

We conducted EFA to control the structural validity of the three scales’ data sets. As shown in Table 4, the outer loadings for all latent constructs fall within the acceptable range to establish indicator reliability. According to Hair et al. (2022), outer loadings should ideally be between 0.7. However, above 0.4 values are considered satisfactory by many scholars. A minor exception is OPAC7, which slightly underperformed relative to the threshold; however, it was retained due to its theoretical significance and negligible impact on overall reliability metrics.

To examine the factor structures of the scales, confirmatory factor analysis (CFA) was conducted based on the factor structure identified through exploratory factor analysis. The results indicated that all three scales demonstrated acceptable to good model fit. Specifically, the normed chi-square (CMIN/DF) values were 2.82 for OPAC, 3.25 for JS, and 2.26 for JP, which fall within commonly accepted ranges for adequate fit (Kline, 2023). This result was not interpreted in isolation because the chi-square test is well known to be highly sensitive to sample size, especially when the sample size exceeds 400 (Stone, 2021). The literature recommends that chi-square should not be treated as the sole basis for judging model fit, but rather considered together with approximate fit indices such as SRMR, CFI, and RMSEA (Alavi et al., 2020; Ockey and Choi, 2015). The CFI values for all three scales exceeded 0.95, the RMSEA values were below 0.06, and the SRMR values were also below the recommended threshold of 0.08, all of which support good model fit (Park and Rosén, 2013). Taken together, these findings provide support for the adequacy of the proposed factor structures of the three scales.

*Convergent validity* assesses the extent to which a construct explains the variance in its indicators. This was evaluated using AVE, where values equal to or above 0.50 indicate that a construct captures at least 50% of the variance in its observed measures (Fornell and Larcker, 1981; Hair et al., 2022). As shown in Table 4, the AVE scores for nearly all constructs exceed this threshold, confirming acceptable convergent validity.

*Discriminant validity* was assessed using the Heterotrait-Monotrait Ratio of Correlations (HTMT), as recommended by Henseler et al. (2015), to verify that the constructs are empirically distinct. All HTMT values fall below the acceptable cutoffs of 0.85 or 0.90, depending on the conceptual similarity of constructs (Franke and Sarstedt, 2019; Hair et al., 2022). These results demonstrate that the constructs exhibit satisfactory discriminant validity.

### 4.2 Reliability analyses

The *internal consistency* of the OPAC, JP, and JS scales was evaluated using Cronbach’s alpha (*α*) and corrected item–total correlations. As shown in Table 5, all *α* coefficients exceed the recommended threshold of 0.70, indicating satisfactory reliability (Hair et al., 2022). Corrected item–total correlation coefficients also surpass the acceptable minimum of 0.30 (Streiner and Norman, 2008; Field, 2024), confirming item consistency within each scale. *Composite reliability* (CR) was also computed to evaluate the internal consistency of each construct. All CR values exceed the 0.70 threshold, indicating strong internal reliability (Hair et al., 2022). According to Hair et al. (2022), CR values between 0.60 and 0.90 are considered satisfactory for reflective models. Together, these findings demonstrate that the OPAC, JP, and JS scales meet accepted psychometric standards and can be considered both valid and reliable for further analysis.

**Table 5:** Outputs of internal consistency and composite reliability.

Scale	JAMOVI-R			SmartPLS		
	Corrected item-total correlation	Total Cronbach (*α*)	CR	Cronbach (*α*)	CR (*rho_a*)	CR (*rho_c*)
OPAC (7 items)	0.790–0.815	0.824	0.931	0.870	0.932	0.902
JP (5 items)	0.811–0.873	0.864	0.863	0.849	0.855	0.891
JS (5 items)	0.804–0.833	0.848	0.936	0.824	0.832	0.869

*n* = 543, CR, Composite Reliability.

Considering the results in Table 5, the output of the internal consistency analysis shows that all Cronbach’s alpha scores are higher than the limit value of 0.7, and the corrected item-total correlation coefficients of the scales are higher than the threshold score of 0.30 (Field, 2024). Additionally, CR values concerning the factors of OPAC, JP, and JS scales are greater than the defined limit value of 0.7. As a result, all reliability-related parameters indicate a notably high level of reliability for the scales. Considering all these findings, it can be concluded that three scales (OPAC, JP, and JS) are valid and reliable. According to Hair et al. (2022), values between 0.70 and 0.90 for Cronbach alphas and values between 0.60–0.90 for composite reliability are typically viewed as satisfactory.

Common method bias (CMB) was assessed using Harman’s Single-Factor Test (HSFT) and the full collinearity variance inflation factor (VIF) approach proposed by Kock (2015). According to Podsakoff et al. (2003) and Fang and Wen (2018), CMB is a possible problem in behavioral research and is the measurement error brought on by social-desirability bias, which is the propensity of survey participants to provide answers that would be seen positively by others (Chang et al., 2010). The outer VIF values ranged from 1.508–2.849 for JP, 1.666–2.459 for JS, and 1.264–2.075 for OPAC, whereas the inner VIF values ranged from 1.000 to 1.205. All values were below the recommended threshold of 3.3, suggesting that neither problematic multicollinearity nor substantial CMB was a concern for the study (Kock, 2015; Hair et al., 2014). In addition, HSFT was conducted using an unrotated exploratory factor analysis, including all items from the multi-item scales. The analysis yielded three factors, with the first factor accounting for 27.00% of the total variance. Since no single factor emerged and the first factor explained less than 50% of the total variance, the findings further suggest that CMB is unlikely to be a serious concern in the present study. However, the findings should still be interpreted with the usual caution applicable to self-reported survey data (Podsakoff et al., 2003).

**Figure 2:**
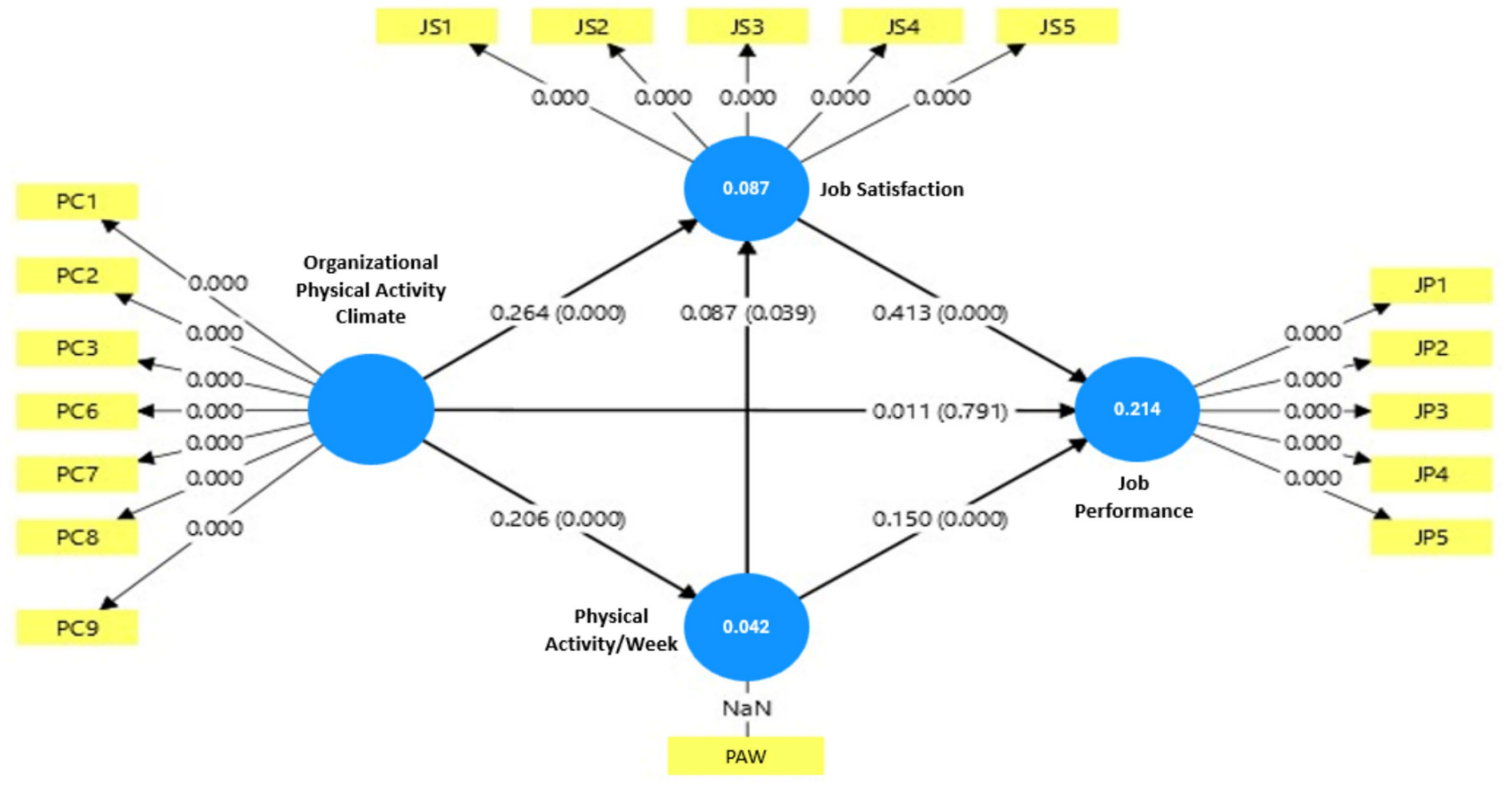
SEM graphical output.

For specifying the relations between independent variables (OPAC, JS, and PA/week), control variables (age, gender, and academic rank), and dependent variables, structural equation modelling (SEM) was performed using the SmartPLS software, while CA were done using JAMOVI-R. All study variables were tested for normality using the Kolmogorov–Smirnov test, and none were found to be normally distributed (*p* < 0.05). Therefore, Spearman’s rho correlation was used. The results of the CA and normality tests are presented in Table 6. Subsequently, the Kolmogorov–Smirnov test of the residuals revealed that they were not normally distributed (*p* < 0.001), violating a key assumption of regression analysis. In light of this and the complexity of the model, SEM was employed using SmartPLS (Figure 2).

**Table 6:** Results of correlation analysis.

	Normality test (ŋ)	1	2	3	4	5	6	7
(1) OPAC	0.002	—						
(2) JS	<0.001	0.259***	—					
(3) JP	<0.001	0.146***	0.389***	—				
(4) PAW	<0.001	0.190***	0.135**	0.164***	—			
(5) Age	0.006	−0.113**	0.127**	0.044	−0.039	—		
(6) Gender	–	0.185***	0.174***	−0.005	−0.008	0.201***	—	
(7) AR	<0.001	−0.106*	0.271***	0.085*	< 0.001	0.287***	0.004	—

Gender coded as 0 = Female, 1 = Male, AR coded as 0 = Asst. Prof., 1 = Assoc. Prof., 2 = Full Prof. *p < 0.05, **p < 0.01, ***p < 0.001.

CA reveals several significant relationships between the variables. Notably, OPAC is positively and significantly associated with both JS (*ρ* = 0.259, *p* < 0.001) and JP (*ρ* = 0.146, *p* < 0.001). Suggesting that when employees perceive a positive OPAC, their JS and JP levels tend to increase. The relationship between OPAC and JP might be explained by the indirect effect, as OPAC is positively correlated with PA per week (PAW) (*ρ* = 0.198, *p* < 0.001), which in turn is positively associated with both JS and JP. A supportive PA climate likely encourages active behaviors, which improve well-being and energy levels, thereby enhancing satisfaction and productivity (Toker and Biron, 2012).

A negative correlation is observed between OPAC and age (*ρ* = −0.113, *p* < 0.01), indicating that younger employees in the sample tend to perceive OPAC more positively. This may reflect generational differences in values and expectations around workplace wellness (Tringali and Aldridge, 2021). Similarly, the positive correlation between OPAC and gender (*ρ* = 0.185, *p* < 0.001) suggests that male employees report higher perceptions of OPAC than females, possibly due to gender differences in PA engagement at work, as men are generally more likely to participate in structured or vigorous PA (Hallal et al., 2012). Moreover, the negative association between OPAC and academic rank (AR) (*ρ* = −0.106, *p* < 0.01) implies that academicians with lower academic ranks perceive a less supportive environment for PA, potentially due to limited institutional influence or reduced access to wellness resources (Çınar and Bavlı, 2014).

The positive correlation between JS and JP (*ρ* = 0.389, *p* < 0.001) is well-documented in the literature, reinforcing the notion that satisfied employees tend to perform better. Expectedly, PAW is positively related to JS (ρ = 0.135, *p* < 0.01), indicating that employees who engage in more PA report higher JS. This could be explained by the psychological benefits of exercise, such as improved mood, reduced stress, and increased energy (Biddle and Asare, 2011). Furthermore, JS is positively associated with Age, Gender, and AR, suggesting that older male employees in higher academic positions tend to report greater JS. This may be attributed to greater job stability, autonomy, and professional recognition that often accompany senior positions (Mgaiwa, 2023; Koirala and Khatiwada, 2024). The correlation between PAW, AR, gender, and age was observed to be negligible and insignificant, suggesting that the academic position, age, and gender of an individual do not affect how much the individual indulges in PA.

PAW also positively influences JP (*ρ* = 0.164, *p* < 0.001), which aligns with prior research showing that physically active employees tend to demonstrate higher work performance due to better physical health, stamina, and mental sharpness. Finally, the positive correlation between JP and AR (*ρ* = 0.085, *p* < 0.05) implies that higher-ranking academicians perform better in their roles, possibly due to accumulated experience, institutional knowledge, or job commitment (Rosario et al., 2025).

The research model (Figure 1) was developed to examine both the direct and indirect effects of OPAC on JP, with JS and PAW as mediators. To distinguish the extent of mediation, the Variance Accounted For (VAF) was calculated, where values below 0.20 indicate no mediation, values between 0.20 and 0.80 indicate partial mediation, and values of 0.80 or above indicate full mediation (Hair et al., 2014). In addition, the magnitude of the beta (*β*) coefficients was interpreted as low at 0.10, moderate at 0.30, and high at 0.50 or above (Kline, 2015).

The findings, as shown in Table 7, confirm multiple indirect pathways. First, OPAC significantly influenced JS (*β* = 0.264, *p* < 0.001), which in turn significantly enhanced JP (*β* = 0.413, *p* < 0.001). The indirect effect of OPAC on JP through JS was also moderately significant (*β* = 0.109, *p* < 0.001), and the corresponding VAF was 90.8%, indicating full mediation for this pathway. Second, OPAC had a significant positive effect on PAW (*β* = 0.206, *p* < 0.001), which also positively predicted JP (*β* = 0.150, *p* < 0.001). The indirect effect of OPAC on JP through PAW was likewise significant (*β* = 0.031, *p* = 0.002) but weak, although the corresponding VAF of 73.8% indicated partial mediation and suggested that this behavioural pathway was comparatively weaker.

**Table 7:** Direct and indirect effects of SEM path analysis.

		Hypotheses	O	M	STDEV	*p*	Result
Direct effects	OPAC → JS	H_1_	0.264	0.269	0.042	0.000	Supported
	OPAC → JP	H_2_	0.011	0.013	0.042	**0.791**	Rejected
	JS → JP	H_3_	0.413	0.415	0.035	0.000	Supported
	OPAC → PAW	H_5_	0.206	0.209	0.044	0.000	Supported
	PAW → JP	H_6_	0.150	0.149	0.037	0.000	Supported
	PAW → JS	H_8_	0.087	0.086	0.042	0.039	Supported
Indirect effects	OPAC → JS → JP	H_4_	0.109	0.112	0.020	0.000	Supported full mediation
	OPAC → PAW → JP	H_7_	0.031	0.031	0.010	0.002	Supported full mediation
	OPAC → PAW → JS → JP	H_9_	0.036	0.036	0.018	0.044	Supported partial mediation

*n* = 543; O, Original path coefficient (β estimate) based on the unbootstrapped sample; M, Mean path coefficient (from bootstrapping); STDEV, Standard deviation of bootstrapped estimates.

In addition, the hypothesized serial mediation pathway was supported, such that OPAC affected JP indirectly through PAW and JS in sequence. Specifically, the indirect effect for the path OPAC → PAW → JS → JP was statistically significant (β = 0.036, *p* = 0.044), accounting for approximately 76.6% of the total effect, indicating partial serial mediation. Showing that OPAC contributes to JP through a chained behavioral and psychological process in which increased PA is associated with higher JS, which in turn enhances JP. However, the magnitude of this serial indirect effect was modest, suggesting that this pathway plays a supplementary rather than central role (Li et al., 2026).

The model explained 8.7% of the variance in JS, 4.2% in PAW, and 21.4% in JP. While these results support the view that OPAC relates to JP through both psychological and behavioral mechanisms, the relatively low R^2^ value for PAW suggests that employees’ PA is also likely shaped by other factors beyond OPAC, such as individual motivation, access to external facilities, and broader cultural norms. Taken together, the findings indicate that JS is the stronger mediating mechanism, whereas the PAW-related pathways, although statistically significant, are comparatively weaker.

## 5 Discussion

### 5.1 Comparison with previous studies

The findings of this study are consistent with earlier research emphasizing the indirect role of PA in improving performance through psychosocial outcomes such as JS. For example, Conn et al. (2009) and Pronk and Kottke (2009) found that PA supports mental health and engagement, which are critical to performance. The mediation role of JS between PA and JP aligns with previous meta-analytical evidence showing that improvements in affective states mediate the relationship between wellness behaviors and workplace performance (Nabe-Nielsen et al., 2015). In contrast to Goetzel et al. (2018) and Proper and van Mechelen (2008), who observed stronger direct links between workplace wellness programs and performance, our study reveals that climate-level influences are better understood when indirect pathways through satisfaction and activity are modeled. Moreover, the low R^2^ value in PAW suggests that, like findings by Jirathananuwat and Pongpirul (2017), organizational support alone may not fully capture all behavioral drivers—personal, environmental, and cultural elements may also play substantial roles. The introduction of OPAC as a measurable construct complements prior work by Coşar et al. (2012), who emphasized the importance of aligning strategic HR practices with health initiatives. Our study provides an individual-level perception-based empirical evidence of this alignment through validated measurement and structural modeling.

### 5.2 Theoretical implications

Theoretically, this study extends the boundaries of organizational climate research by proposing and validating OPAC as a distinct individual perception-based climate-level construct. The results underscore the role of OPAC in shaping both behavioral (PAW) and attitudinal (JS) employee responses, which subsequently influence job performance. The dual mediation model supported here reflects three major theoretical perspectives in organizational behavior. First, the Job Demands–Resources Model conceptualizes OPAC as a job resource that supports employee well-being and buffers against strain. Second, Social Cognitive Theory helps explain how environmental features like OPAC enhance self-efficacy and foster health-supportive behaviors such as regular PA. Third, Conservation of Resources Theory posits that OPAC facilitates the accumulation and preservation of valuable personal resources—such as energy, resilience, and emotional balance—which are critical to sustained performance. Fourth, and importantly, this study contributes to Climate Theory by demonstrating that individual level perceptions of organizational support for PA form a distinct and measurable climate. By extending climate theory to encompass health-oriented climates like OPAC, the findings highlight that climate constructs are not limited to traditional domains such as safety or service but can also encompass proactive health promotion, thereby broadening the scope and application of climate research in organizational behavior. Together, these theoretical frameworks provide a comprehensive explanation for how OPAC influences job performance indirectly through behavioral and psychological mechanisms.

### 5.3 Practical implications

Practically, the findings highlight that fostering a supportive OPAC is not only beneficial for employee well-being but also serves as a strategic tool for enhancing job performance. Interventions that promote PA should be designed to also enhance JS, given their stronger mediating influence. Managers are encouraged to align wellness programs with organizational goals, use visible prompts and social incentives, and tailor initiatives to the specific preferences and demographics of employees. Although OPAC’s association with PAW was statistically significant, the relatively low explained variance in PAW (*R^2^* = 0.042) suggests the need to explore other influences, such as workload, motivation, or physical accessibility.

## 6 Conclusion

This study contributed to the literature by introducing and validating the concept of OPAC and examining its effects on JS, PAW, and JP. The findings revealed that OPAC significantly enhanced both JS and PAW. While OPAC did not have a direct effect on JP, its influence was fully mediated by JS and PAW. Additionally, JS partially mediated the relationship between PAW and JP, indicating that PA contributed to performance through both direct and indirect pathways. Notably, JS had a stronger effect on JP than PAW, highlighting the dominance of psychological mechanisms in performance outcomes. The model explained 8.7% of the variance in JS, 4.2% in PAW, and 21.4% in JP.

Despite its contributions, this study has several limitations. First, the data were collected using a cross-sectional design, which limits the ability to infer causality. While PLS-SEM via SmartPLS was employed to explore complex mediation relationships, making the approach robust in theory development and less sensitive to sample distribution and size, it does not fully overcome the limitations inherent in non-longitudinal data. Future research utilizing longitudinal or experimental designs would allow for stronger causal inferences and temporal validation of the observed relationships. Second, the sample was restricted to academic professionals in Istanbul, which may limit the generalizability of findings to other sectors or geographic contexts. Third, as is common in behavioral research, reliance on self-reported measures introduces the potential for response biases such as social desirability. To mitigate concerns related to CMB, we conducted the HSFT and the full collinearity VIF approach proposed by Kock (2015). As none of the single factors accounted for the majority of the variance and all the reported VIF values were well below the threshold limits, CMB was deemed not to be a major concern for this study. However, the results should still be interpreted with appropriate caution, as self-reported data are inherently subject to some degree of response bias. Moreover, the relatively low variance explained for PAW suggests that OPAC plays a supportive, rather than an exclusive, role in promoting employees’ PA. Additionally, university names were not collected for ethical reasons, as universities did not wish to be identifiable in the study. Although this approach supported respondent openness and reduced socially desirable responding, it also prevented the assessment of OPAC as a higher-level organizational climate construct through aggregation statistics or multilevel analysis. OPAC was therefore treated as an individual-level psychological climate perception. Lastly, despite its practical utility, the single-item measure of PA is a limitation. Because it captures only self-reported frequency, it does not fully reflect the multidimensional nature of physical activity. Therefore, findings related to physical activity should be interpreted with caution.

Future research should consider longitudinal designs to examine how OPAC evolves over time and affects performance outcomes dynamically. Researchers may also explore moderating factors, such as leadership style, organizational support, and individual health status, to understand under what conditions OPAC is most effective. Moreover, future research is encouraged to administer the questionnaire across identifiable organizations and collect data that allow respondents to be nested within their institutional settings. This would make it possible to compare OPAC across organizations, evaluate within-organization agreement, and conduct aggregation as well as multilevel analyses, thereby providing a stronger basis for assessing OPAC as a higher-level organizational climate construct in addition to an individual-level psychological climate perception. Additionally, cross-cultural and sectoral studies could assess how cultural norms and different organizational settings influence the perception and impact of a supportive OPAC. Finally, integrating objective health metrics or wearable activity data could enhance the validity of PA measurements and clarify behavioral mechanisms more precisely.

## Data Availability

The original contributions presented in the study are included in the article/Supplementary material, further inquiries can be directed to the corresponding authors.
