# Supplementary material for: Beyond wellness: modeling the impact of organizational physical activity climate on job performance through psychological and behavioral pathways
Source: Front Psychol. 2026 May 20;17:1817693. doi: 10.3389/fpsyg.2026.1817693 (PMC13229741; doi:10.3389/fpsyg.2026.1817693)
Supplement: Supplementary file 1 [file Supplementary_file_1.docx]

Supplementary Materials

Table S.1: Descriptives.

| Variable | | *µ* | *σ* | Variable | | f | % |
| --- | --- | --- | --- | --- | --- | --- | --- |
|  | OPAC | 2.47 | 0.91 | Regular PA | Yes | 294 | 0.541 |
|  | JS | 3.43 | 0.89 |  | No | 249 | 0.459 |
|  | JP | 5.84 | 0.97 | PAW | 0 | 128 | 23.6 |
|  | Age | 47.8 | 7.88 |  | 1 | 55 | 10.1 |
| Variable | | f | % |  | 2 | 95 | 17.5 |
| Gender | Female (0) | 236 | 43.4 |  | 3 | 118 | 21.7 |
|  | Male (1) | 307 | 56.6 |  | 4 | 75 | 13.8 |
| Academic rank | Full Professor | 88 | 16.2 |  | 5 | 43 | 7.9 |
|  | Associated Professor | 200 | 36.8 |  | 6 | 18 | 3.3 |
|  | Assistant Professor | 255 | 47.0 |  | 7 | 11 | 2.0 |
| Happiness with supervisor | Yes | 298 | 0.548 |  |  |  |  |
|  | No | 251 | 0.452 |  |  |  |  |

*Note. n*=543, f: frequency, PA: Physical Activity, HWS: Happiness with Supervisor, OPAC: Organizational Physical Activity Climate, JS: Job Satisfaction, JP: Job Performance, PAW: Physical Activity/Week.

Table S.2: Outputs of exploratory factor analysis and outer loadings.

| Variable | Factor desc. statistics | | EFA (JAMOVI-R) | | | | SmartPLS | | HTMT–Matrix | | |
| --- | --- | --- | --- | --- | --- | --- | --- | --- | --- | --- | --- |
|  | Mean | SD | Factor loadings | (%) Variance | KMO test | Bartlett’s test (*p*) | Outer loadings | AVE | 1 | 2 | 3 |
| OPAC  (7-item) | 2.47 | 0.911 | 0.675-0.755 | 48.9 | 0.835 | 0.001 | 0.599-0.772 | 0.648 | - |  |  |
| JP  (5-item) | 5.84 | 0.966 | 0.705-0.878 | 66.1 | 0.848 | 0.001 | 0.764-0.854 | 0.621 | 0.451 | - |  |
| JS  (5-item) | 3.47 | 0.850 | 0.741-0.829 | 62.4 | 0.801 | 0.001 | 0.749-0.820 | 0.489 | 0.166 | 0.324 | - |

*Note. n*=543, HTMT: Heterotrait-Monotrait Ratio.

Table S.3: Outputs of internal consistency and composite reliability.

| Scale | JAMOVI-R | | | SmartPLS | | |
| --- | --- | --- | --- | --- | --- | --- |
|  | Corrected item-total correlation | Total Cronbach (*α*) | CR | Cronbach  (*α*) | CR (*rho_a*) | CR  (*rho_c*) |
| OPAC (7 items) | 0.790-0.815 | 0.824 | 0.931 | 0.870 | 0.932 | 0.902 |
| JP (5 items) | 0.811-0.873 | 0.864 | 0.863 | 0.849 | 0.855 | 0.891 |
| JS (5 items) | 0.804-0.833 | 0.848 | 0.936 | 0.824 | 0.832 | 0.869 |

*Note. n*=543, *CR*: Composite Reliability.
